# Accuracy and safety of freehand vs. end-on fluoroscopic guided drill-hole placement in canine cadaveric thoracic, lumbar and sacral vertebrae

**DOI:** 10.3389/fvets.2024.1419521

**Published:** 2024-06-13

**Authors:** Colin J. Driver, Victor Alves Nores, Heidi Thatcher, Maria Navarro-Carrillo, Jeremy Rose

**Affiliations:** Lumbry Park Veterinary Specialists, CVS Referrals, Alton, United Kingdom

**Keywords:** vertebral column, veterinary spinal surgery, implant accuracy, implant safety, fluoroscopy

## Abstract

**Objective:**

To develop and evaluate the safety and accuracy of an open, end-on fluoroscopic guided (EOFG) drill hole position technique in canine cadaveric spinal surgery, in comparison to a traditional free-hand (FH) drilling technique.

**Study design:**

Cadaveric comparison study.

**Animals:**

Canine cadaveric vertebral columns (*n* = 4).

**Methods:**

Computed tomography (CT) scans were performed for *in-silico* planning. Ideal implant purchase depth and angulations were determined from previously published data. Plans for end-on fluoroscopic guided drill holes included angled reconstructions in thick slab mode to mimic fluoroscopic images. Following surgical preparation of T8 to S2, holes were drilled by one of two experienced surgeons randomized evenly by operated side, surgeon, and technique. C-arm fluoroscopy was utilized for the end-on technique. CT was repeated after the procedures. Safety was determined categorically using a modified Zdichavsky classification and “optimal” placement was compared between techniques. Continuous data for drill-hole accuracy was calculated as angle and depth deviations from the planned trajectories. Data sets were analyzed at both univariable and multivariable levels with logistic regression analysis.

**Results:**

Drill hole safety was categorized as optimal (modified Zdichavsky classification 1) in 51/60 (85%) of drill holes using EOFG and 33/60 (55%) using FH (*P* < 0.001) techniques. There were no “unsafe” holes (modified Zdichavsky classification 3a). Optimal drill hole placement was significantly associated with the EOFG technique and use of the largest cadaver, and was significantly less likely within the thoracic region. Mean angle and depth deviations were significantly lower with the EOFG technique. Angle deviations were significantly lower for EOFG in the lumbar region, whereas bone purchase deviations were significantly lower for EOFG in both the thoracic and lumbar regions. The mean time taken to drill the hole was significantly longer for the EOFG technique.

**Conclusion:**

Optimal drill hole placement was significantly more likely with the EOFG technique and improved the accuracy of bone purchase in the thoracic region.

**Clinical significance:**

The EOFG technique shows promise for translation into a clinically setting, potentially improving implant purchase and therefore stabilizing construct strength, whilst potentially reducing the likelihood of neurovascular injury and need for surgical revision.

## 1 Introduction

Metallic instrumentation is applied surgically for a range of spinal disorders of dogs that require vertebral stabilization, including trauma resulting in fracture and/or luxation (1), congenital malformation (2), infection resulting in diskospondylitis or osteomyelitis (3), and neoplasia (4). Instrumentation is typically bilateral, although unilateral fixation has been described (5, 6). Potential techniques include the use of pins or screws and polymethylmethacralate (PMMA) bone cement (2, 7, 8), locking bone plates (9), clamp rod internal fixator (10), external fixator (11), and polyaxial pedicle screw and rods (12). Preferred insertion points and trajectories have been published (13) with the main target for bone purchase being the vertebral bodies, although the vertebral pedicles are another attractive option particularly in the caudal lumbar spine (14).

The safe and accurate placement of vertebral implants is technically challenging due to the variable morphology of canine vertebra and associated important neurovascular structures (15). In addition, important soft tissue structures adjacent to thoracic vertebra (right azygos vein, the aorta, the pleura, lungs, spleen, liver, esophagus, sympathetic trunk, and the thoracic canal) are at risk of injury (13). Techniques assessed for safety and accuracy include “free-hand” placement, which is the use of anatomical landmarks and trajectories determined from pre-operative cross-sectional imaging (16), fluoroscopic guidance (11), use of patient-specific 3D-printed resin drill guides (17, 18), and intra-operative neuronavigation (19). Aside from accuracy, each technique has relative advantages and disadvantages, including equipment availability, planning time, and cost.

Despite the ubiquity of image-intensification for assisted implant placement in human spinal surgery, reports of intra-operative spine fluoroscopy in dogs have been limited to closed percutaneous placement of implants at a fixed angle of 30° (11) and closed positioning of spinal external skeletal fixators (20). The relative paucity of reports may reflect the difficulty in imaging the more angled/less cuboidal canine vertebral anatomy. Recently, end-on fluoroscopy has been shown to be accurate in determining the position of implants relative to the thoracolumbar vertebral canal in canine cadavers, which may represent a suitable technique in preference to computed tomography (CT) during post-operative assessment (21). End-on fluoroscopy represents an interesting alternative to free-hand placement in improving implant accuracy given C-arm units are often present in veterinary clinics for other interventional procedures and could be adapted for use at varying angles relative to the long axis of the canine vertebral column, adapting the exposure angle according to regional morphology.

Our aim was to develop and describe the safety and accuracy of an open, end-on fluoroscopic guided drill hole position technique (EOFG) in canine cadaveric spinal surgery, in comparison to a traditional free-hand technique (FH), assessed by post-procedural CT. We hypothesized that EOFG is safe and more accurate than FH in all spine regions assessed and that they are most significantly different for the thoracic, rather than lumbar or sacral spine.

## 2 Materials and methods

### 2.1 Study design

This study was a cadaveric methods comparison study (FH vs. EOFG). The study subjects were four dog cadavers weighing >20 kg, euthanized for reasons unrelated to spinal disease, with no previous clinical history of spinal disease. Use of the cadavers was approved by written owner consent following institutional ethical approval of the study. A power analysis for sample size was initially conducted, based on a study with similar methodology assessing implant insertion accuracy (18), suggesting that a comparison of expected mean differences would require a minimum of 59 drilled holes per technique to have 95% confidence and 80% power. We therefore aimed to perform the two techniques on 15 vertebrae (one technique for each side) from four cadavers (T8 to T13, L1 to L7, and S1 and S2) for a total of 120 drilled holes (60 for FH and 60 for EOFG).

### 2.2 Subject preparation and technique planning

Cadavers were utilized for the study within 24 h of euthanasia. A planning CT (Siemens Somatom Scope, 16 slice) was first performed with the cadaver in sternal recumbency. The helical scan protocol encompassed images from the first thoracic vertebra to the first coccygeal vertebra. Acquisition parameters included a slice thickness of 0.75 mm, a pitch of 1, a matrix size of 512 × 512 and utilization of a bone kernel for image processing. *In-silico* planning for both techniques was performed in commercially available DICOM (digital imaging and communications in medicine) viewing software (OsiriX MD Dicom Viewer Pixmeo Sarl^®^, version 13.0.1, Geneva, Switzerland) with 3D multi-planar reconstruction (MPR) perpendicular to the long axis of the vertebral column. Region of interest (ROI) tools for measuring angle and distance were used.

**Figure 1:**
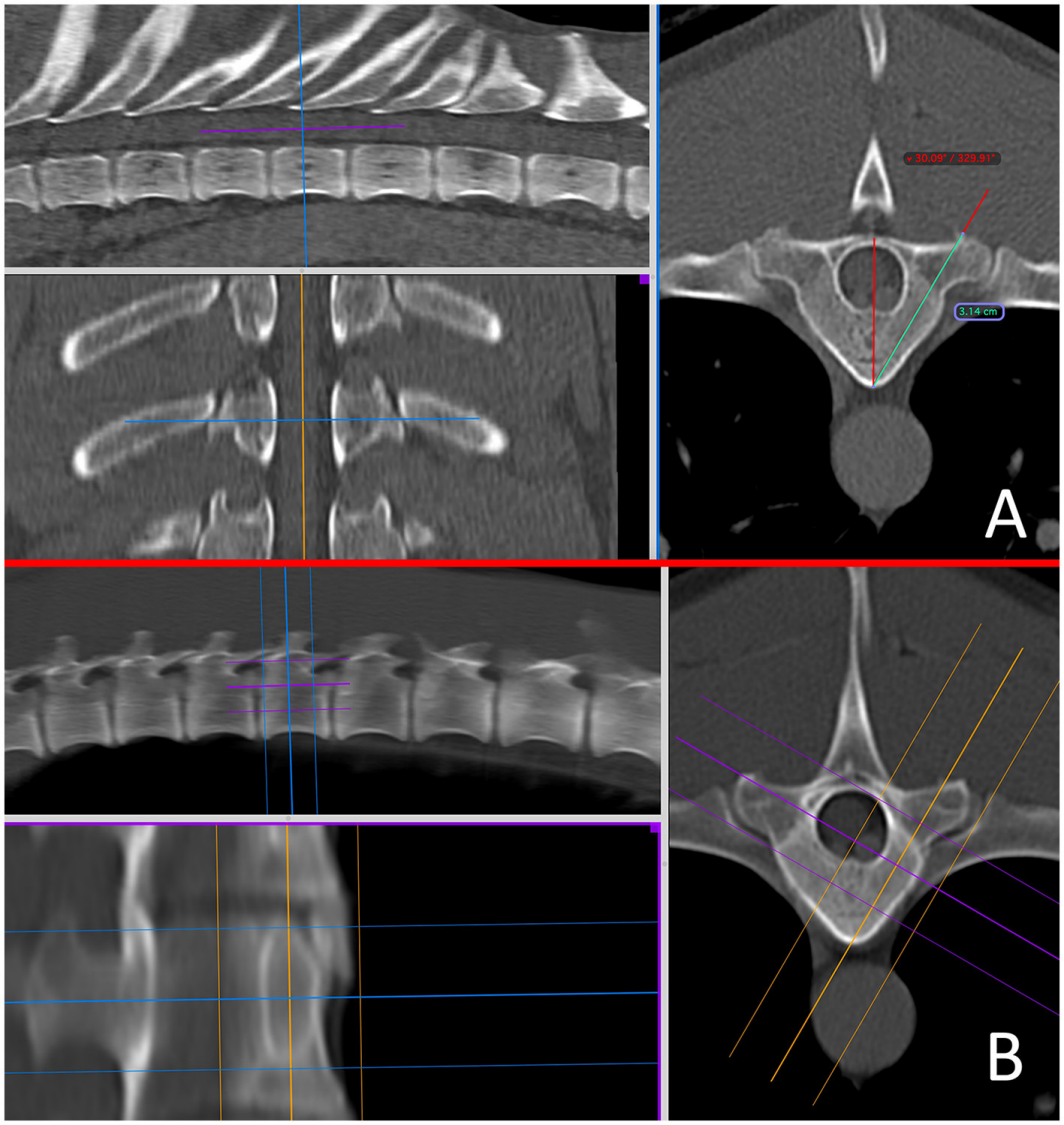
Pre-operative *in-silico* planning from CT scans in Osirix using multi-planar reconstructions in a bone window, for the free hand technique **(A)** and the end-on fluoroscopic guidance technique **(B)** at the level of T10. A 15–20 mm slice thickness is used to mimic the fluoroscopic image.

The planning CT was first used to confirm that there were no incidental diseases affecting vertebral morphology (malformation, osteolytic or productive lesions related to suspected spinal neoplastic, inflammatory, or infectious disease). Plans were then produced for all 120 drilled holes, as follows:

For FH, ideal implant insertion points and angulations were determined from previously published data (13) and surgeon preference, then recorded for future reference (Figure 1A). Insertion points were typically planned to be identified from orientation points such as the base of the accessory process, the tubercle of the ribs, the distance from the articular processes or the transverse processes. The preferred angle of insertion was determined relative to the mid-point of the vertebral lamina and spinous process, aiming for a safe column (mid-way between the lateral margin of the vertebral pedicle or body and the vertebral canal) aiming for maximal implant bone purchase.

For EOFG, ideal insertion points and angulations were similarly determined, but were subsequently assisted using 3D-MPR in “thick slab mode” adjusting the mean slice thickness to between 15 and 20 mm (interval 0 mm), orientated to the angle of implant insertion, such that the reconstructed image in a dorsal oblique plane would mimic the desired appearance of intra-operative fluoroscopy (Figures 1A, B).

### 2.3 Cadaveric technique

Cadavers were prepared surgically with the paraspinal musculature reflected from the vertebrae of the mid thoracic to the sacral region and held with Gelpi self-retaining retractors. Care was taken to remove all soft-tissue attachments to the bony orientation points. All holes were drilled by one of two experienced spinal surgeons according to the technique plans, randomized evenly by operated side (left or right), surgeon (CD or JR), and technique (FH or EOFG). The time taken to drill each hole (from initial assessment of insertion position to penetration of the trans cortex) was recorded.

**Figure 2:**
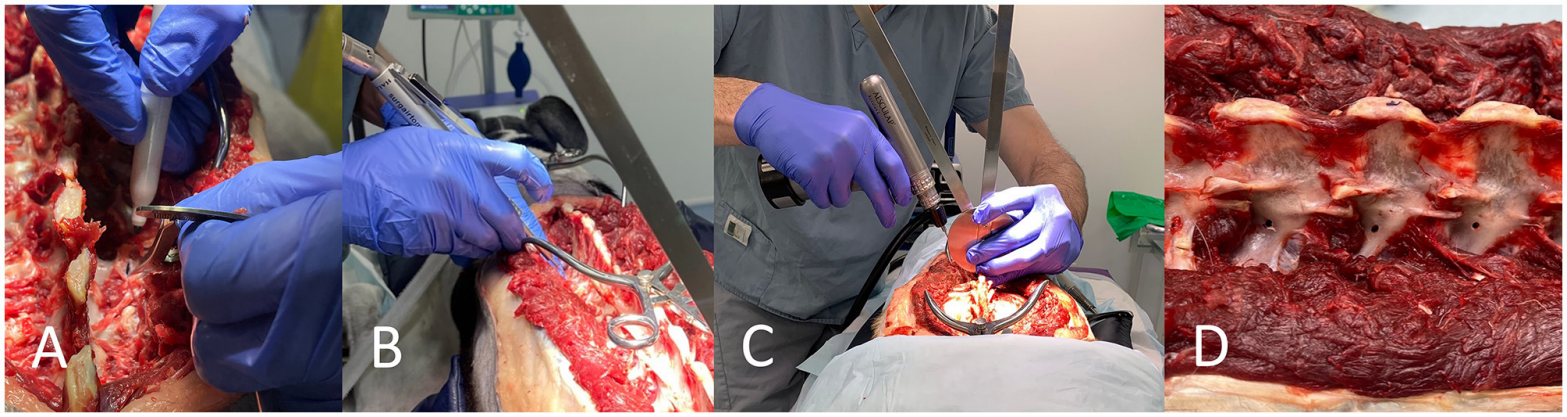
Free hand technique. The insertion point is determined from *in-silico* planning with the assistance of bony landmarks and tools such as a pen and caliper **(A)**. The insertion point is decortified with a 2 mm burr **(B)**. A goniometer is used in close proximity to the vertebrae and the drill for drilling along the planned angle **(C)**. Drill holes can be seen **(D)**.

For FH, the insertion point was determined from the pre-operative plans and bony orientation points, then decortified with a 2 mm high-speed burr. The insertion angle was then determined using a goniometer held close to the patient and the drill. A 2.0 mm (cadaver weighing 20–25 kg) or 2.5 mm diameter (25 kg+) drill bit (Veterinary Instrumentation) was then used to drill a bi-cortical hole (Figure 2).

**Figure 3:**
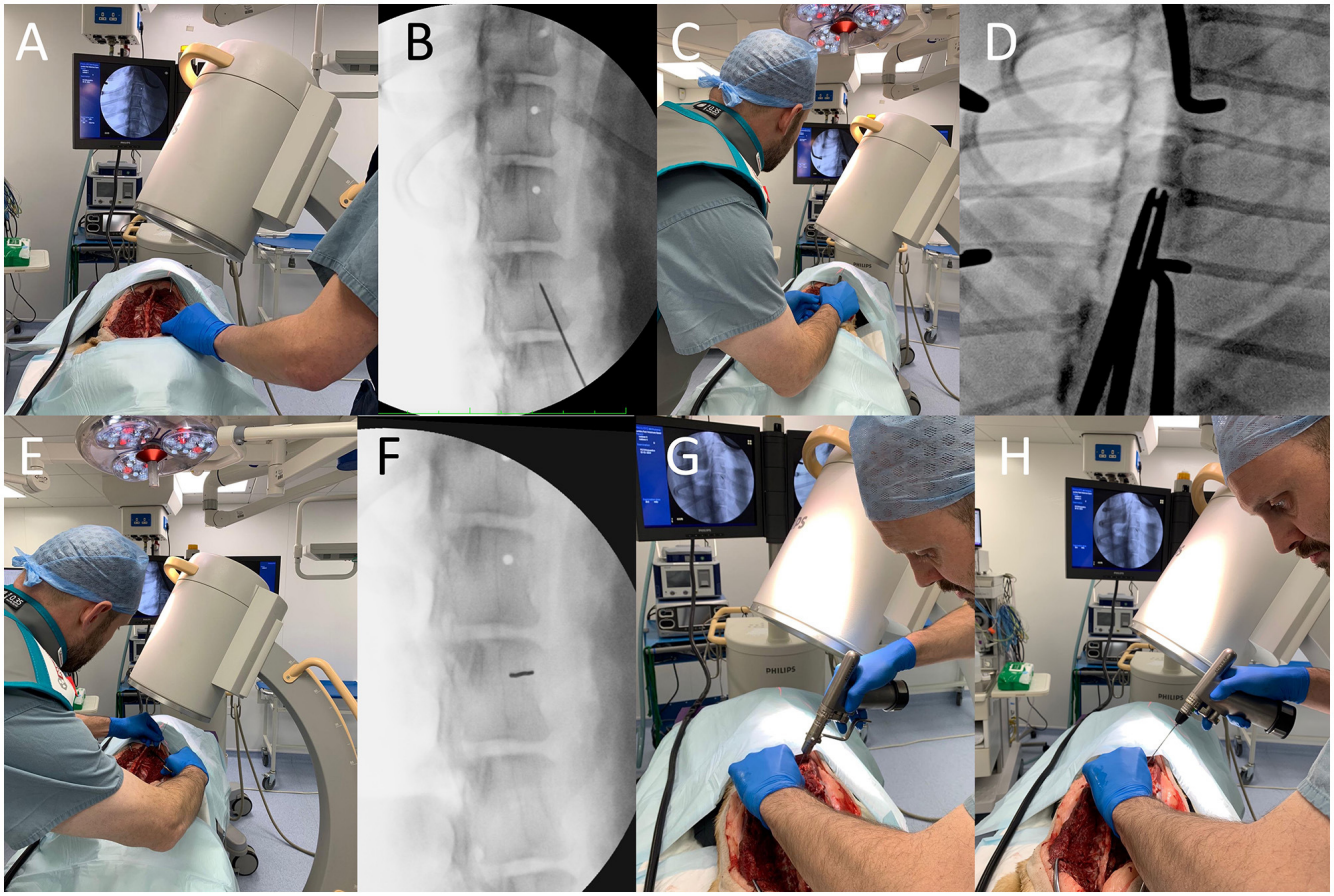
End on fluoroscopic guidance technique. First, the implant insertion point is estimated based on *in-silico* plans and pointed to with a k-wire held with long handled forceps **(A)**, the position of which is confirmed with video-fluoroscopy **(B)**. This insertion point is then decortified with a 2 mm high-speed burr and the k-wire is re-orientated along the primary beam **(C)** such that it has as small a cross-sectional area as possible **(D)**. The k-wire is then firmly inserted 2–3 mm by hand **(E)** and the position is again checked **(F)**. The C-arm detector is “slid” sideways away from the surgical field, so that the k-wire can then be inserted through the cancellous bone until it contacts the trans cortex **(G)**. A cannulated drill-bit is then used to drill the hole and penetrate the trans cortex **(H)**.

For EOFG, local radiation safety rules concerning fluoroscopy were obeyed, including the use of personal and finger ring dosimeters. C-arm (Philips BV Pulsera Mobile C-arm Unit) angulation was set according to the *in-silico* plan and was orientated to the approximate insertion point on the vertebral body or pedicle using its guidance laser. X-ray projection parameters varied according to the physical properties of each cadaver (voltage 55–75 kV, collimation 14 cm, continuous and fluoroscopic mode at 25 frames per second). A 1.1 mm k-wire, held by long-handled forceps, was used to identify the ideal insertion point under fluoroscopic guidance. This mark was then decortified, using a 2 mm high-speed burr. The k-wire could then be firmly inserted by hand 2–3 mm into the cancellous bone such that it was gripped along the approximate insertion trajectory. Intermittent fluoroscopic projections were used to align the k-wire to its smallest cross-sectional opacity, such that it is aligned along the trajectory of the x-rays (i.e., is seen “end-on”). When satisfied, the surgeon used a motorized pin driver to drive the pin through into the cancellous bone to engage with the trans-cortex, such that its trajectory could not easily be modified. In some instances, it was necessary to “slide” the C-arm sideways (maintaining the same trajectory and distance from the cadaver and operating table) away from the region of interest to create space for the surgeons hand and the drill. After this, a cannulated drill bit (2.0 or 2.5 mm) was used to drill the bi-cortical hole, with penetration of the trans cortex determined from tactile feedback (Figure 3). In large dogs (cadaver weighing > 35 kg) the cannulated drill bit was used to drill the cis-cortex and cancellous bone, then once the k-wire had been removed, a non-cannulated drill bit could instead be inserted into the hole and used to penetrate the trans cortex (this was helpful to prevent the k-wire becoming stuck in the cannulated drill bit).

When all holes were drilled, the wound was closed, and the cadaver was transferred for repeat CT of the spine with the position, acquisition protocol and field of view to match the previous scans.

### 2.4 Image evaluation

**Figure 4:**
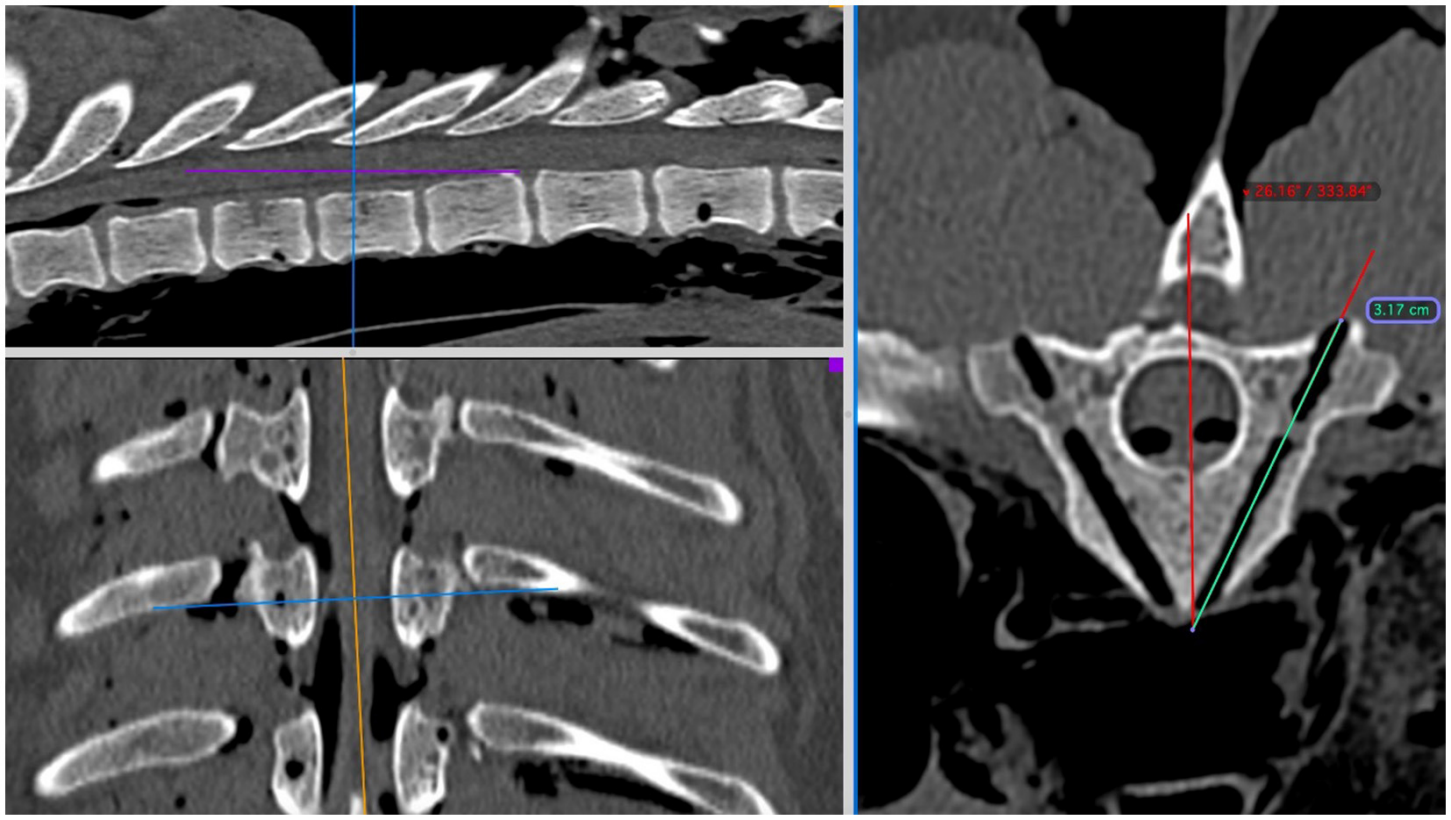
Post-procedural assessment of CT scans in Osirix using multi-planar reconstructions in a bone window at the level of T9. The region of interest tools for angle and length can again be used to assess deviations from the pre-procedure plans.

All CT images were evaluated by one of the authors (CD) with Osirix using 3D-MPR in a bone window using ROI tools as previously described (Figure 4). Continuous data for drill-hole accuracy was determined in two ways. Firstly, “angle deviation” (in degrees) from the planned angled trajectory of insertion was determined and recorded. Secondly, “depth deviation” (in mm) below the planned insertion depth was recorded (representing the accuracy of obtaining the minimum preferred bone purchase). Where this value was negative (i.e., greater bone purchase achieved) it was recorded as zero.

Safety was determined categorically using a modified Zdichavsky classification (22) (a validated scoring system for pedicle screw placement in the human spine by using defined criteria), as previously described elsewhere (16) but adapted to include the sacrum according to the same principles.

### 2.5 Statistical analysis

Data sets were analyzed according to all data, data by spinal region (thoracic vs. lumbar vs. sacral), and data by surgeon. All data sets were assessed for normal distribution. Statistical significance was set at *P* < 0.05.

In determining drill-hole safety, modified Zdichavsky grade 111a holes were categorized as “unsafe” and all other grades categorized as “safe.” Modified Zdichavsky grade 1 holes were categorized as “optimal” and all other grades categorized as “suboptimal.” Differences in safety category of the two methods were incorporated into multivariable logistic regression analysis to take cadaver number, technique, and spinal region into account. In determining drill-hole accuracy, the differences in angle and depth deviations between the two techniques were compared in a paired vertebrae analysis at the univariable level using either paired *t*-tests (if normally distributed) or a Wilcoxon signed rank test (non-normally distributed) first considering all drill-holes and then by spinal region.

The differences in time taken between the two techniques will be compared in a paired vertebrae analysis using either paired *t*-tests (if normally distributed) or a Wilcoxon signed rank test (non-normally distributed).

## 3 Results

Four canine cadavers were used for the study of the following breeds and body weights: Greyhound (35 kg), German Shepherd dog (GSD, 38 kg), and two crossbreeds (27 and 23 kg). Mean age was 6.5 years (range: 5–8 years), and there were two male and two female dogs. None of the vertebral columns had radiographic evidence of bony changes. One hundred and twenty holes were drilled as planned, 60 for each technique.

**Figure 5:**
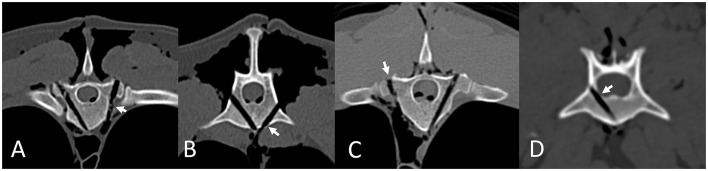
Drill hole errors according to the modified Zdichavsky grade, assessed using post-procedure CT scans in Osirix using multi-planar reconstructions in a bone window. Errors are highlighted by short arrows. Type 2b errors in the thoracic **(A)** and lumbar **(B)** spine. Type 3b error in the thoracic spine **(C)**. Type 2a error in the lumbar spine **(D)**.

Drill-hole safety data is summarized in Table 1. There were no “unsafe” holes drilled using either technique in the study. Type 2b and 3b errors were most common in the thoracic spine and with the FH technique (Figure 5). Overall, drill hole placement was categorized as optimal in 51/60 (85%) of drill holes using EOFG and 33/60 (55%) using FH (*P* < 0.001). Percentages for optimal drill hole placement between the two techniques (EOFG and FH) were lower in the thoracic (71 and 38%, respectively) in comparison to the lumbar (93 and 61%) and sacral (100 and 88%) segments. In the multivariate analysis, optimal drill hole placement was significantly associated with the EOFG technique (*P* < 0.001, OR = 7.34, 95% CI: 2.73–22.29) and the largest (GSD) cadaver (*P* = 0.002, OR = 16.37, 95% CI: 3.23–131.4), whereas optimal placement was less likely within the thoracic region (*P* = 0.007, OR = 0.24, 95% CI: 0.08–0.65).

**Table 1:** Summary of drill-hole safety data (EOFG, end-on fluoroscopic guidance technique; FH, free hand technique).

**Region**	**Technique**	**Modified Zdichavsky grade**					**Optimal %**
		**1**	**2a**	**2b**	**3a**	**3b**	
ALL	EOFG	51	2	7	0	0	85
ALL	FH	33	3	13	0	11	55
Thoracic	EOFG	17	0	7	0	0	71
	FH	9	0	8	0	7	38
Lumbar	EOFG	26	2	0	0	0	93
	FH	17	3	5	0	3	61
Sacral	EOFG	8	0	0	0	0	100
	FH	7	0	0	0	1	88

Accuracy data is summarized in Table 2. Overall, there was a significant difference in mean (± SEM) angle deviation between the EOFG (3.30° ± 0.46°) and FH (5.07° ± 0.51°) techniques (*P* = 0.009). There was also a significant difference in mean bone purchase deviation between the EOFG (1.07 ± 0.32 mm) and FH (3.60 ± 0.68 mm) techniques (*P* < 0.001). In the paired univariate analysis by region, mean angle deviations are significantly lower for EOFG in the lumbar region (*P* = 0.013) whereas mean bone purchase deviations are significantly lower for EOFG in both the thoracic (*P* = 0.017) and lumbar (*P* = 0.007) regions.

**Table 2:** Summary of drill-hole accuracy data including results of univariable analysis (EOFG, end-on fluoroscopic guidance technique; FH, free hand technique).

**Variable**	**Region**	**Technique**		***P*-value**
		**EOFG**	**FH**	
Angle deviation^a^	ALL	3.30 (0.46)	5.07 (0.51)	0.009^*^
Purchase depth deviation^b^	ALL	1.07 (0.32)	3.60 (0.68)	< 0.001^*^
Angle deviation^a^	Thoracic	4.17 (0.94)	6.21 (0.95)	0.15
	Lumbar	2.68 (0.40)	4.29 (0.64)	0.013^*^
	Sacral	2.88 (1.51)	4.38 (1.02)	0.472
Purchase depth deviation^b^	Thoracic	1.71 (0.65)	5.33 (1.25)	0.017^*^
	Lumbar	0.11 (0.08)	2.00 (0.64)	0.007^*^
	Sacral	2.50 (1.05)	4.00 (2.32)	0.368

^a^Angle deviation in mm (mean ± standard error of mean).

^b^Purchase depth variation in degrees (mean ± standard error of mean).

Asterisk denotes statistical significance (P-value set at < 0.05).

There was a significant] difference (*P* < 0.001) in mean (± SEM) time taken to drill the hole between the EOFG (4 min 39 s ± 17 s) and FH (1 min 52 s ± 7 s) techniques.

## 4 Discussion

To date, intra-operative fluoroscopic guidance for canine spinal instrumentation has been limited to percutaneous pin positioning at a fixed angle (11). This contrasts with human spinal surgery, where fluoroscopic assistance for transpedicular screw implantation has represented the normal methodology for several decades when assessing vertebral canal or lateral pedicle wall breaches in the thoracic (23) and lumbar (24) spine. This difference might reflect the relatively cuboidal shape to human thoracolumbar vertebrae (meaning implantation is more easily performed in the anterior-posterior plane) and the lack of morphologic variance that dogs demonstrate between breeds.

The first description of EOFG aimed to predict implant position in relation to the vertebral canal as an alternative method of determining safe implantation to post-operative CT (21). Our purpose was to develop and describe a similar technique for open intra-operative use that could overcome some of the morphologic challenges in dogs and allow for pin or screw implantation. In the current study, EOFG for drilling canine cadaveric vertebra was found to be safe when employed by experienced spinal surgeons, avoiding any vertebral canal breaches. For experienced surgeons, FH is also a safe technique, but EOFG was found to be significantly more accurate with significantly fewer suboptimal drill hole placements. Type 2b and 3b errors were most common in the thoracic spine and with FH technique; this suggests experienced spinal surgeons tended to err on the side of safety, at the cost of reducing accuracy. Improved accuracy with EOFG might have a positive influence on post-operative spinal stability through strength of the implant construct with improved bone purchase.

In comparison to existing studies on the accuracy of fluoroscopic techniques, our findings were consistent with those of Wheeler et al. (11), where fluoroscopy significantly improved the mean bone purchase in comparison to an open technique except for T10 and T11 (11). In the human literature, slight lumbar vertebral canal breaches may occur more often than expected (24). There are no similar veterinary studies examining safety for fluoroscopic techniques. 5/120 holes were found to contact the cortex of the lumbar vertebral canal (type 2a error; 3 with FH and 2 with EOFG) whereas there were no “unsafe” vertebral canal breaches (type 3a error). Thoracic vertebral canal breach is also uncommon in human fluoroscopic guided spine surgery, with >2 mm breaches occurring in < 1% of implants in one study (23). It was significantly more common to see slight thoracic lateral pedicle wall breaches (68% of implants) in the same study (23). This is similar to our findings, where type 2b and 3b errors were more common in the thoracic spine (71% of all of those error classes occurring in the thoracic region) relative to other regions. Considering those errors in the thoracic region, EOFG appeared particularly useful, given 68% of type 2b and 3b errors were made with FH technique. This is reflected by the significant improvements in angle and depth deviations, with EOFG drill-holes being more likely to offer better bone purchase and potentially resistance to fatigue, pull-out, and implant failure. This may be related to the fact that the thoracic vertebral pedicle is a narrower target in comparison to the lumbar vertebral body which is easier to access without the presence of the ribs, intercostal and other critical adjacent soft-tissues.

Concerning FH techniques, publications vary somewhat in methodology based on the experience of the surgeon (25), whether a pedicle probing technique is used (26) and whether a metallic implant is inserted rather than assessing a drilled hole. This results in estimates of optimal FH implant placement varying from 51.68 to 87.5% (25, 26) and makes direct comparison to the current study challenging, where modified Zdichavsky grade I was assigned to 55% of drilled holes using the FH technique. Type 2b and 3b errors were most common, resulting in significantly greater angle deviations and lower bone purchase in comparison to EOFG. In the human literature, free hand implantation of pedicle screws resulting in violation varies in frequency from 15 to 41% (22, 27, 28).

There has been increasing interest in the use of patient-specific three-dimensional printed resin drill guides for canine spine surgery which offer a high degree of safety and accuracy (17, 18, 29–32), including in comparison to FH techniques in cadavers (18, 26). However, there are several disadvantages to this technique, including delay in time (potentially rendering it not suitable for vertebral fracture luxation), increased cost, and inconsistent availability of software and hardware. The technique also requires an open approach and may require more extensive soft-tissue exposure to accommodate guide tunnels. As drilling holes with the EOFG technique took significantly more time than FH, it is also likely that drill guides would save intra-operative time in comparison to EOFG.

Although the technique was planned from cross-sectional imaging in this study, it has the potential to be used instead of CT, particularly when this modality is not readily available. This, however, would assume that the surgeon and assisting radiographer have sufficient experience with the expected anatomy and there is no considerable variance in vertebral morphology that would limit published references for implant trajectory. Further clinical studies would be required to investigate this possibility.

A significant limitation to the use of fluoroscopy is the risk of radiation exposure to surgeon and patient. In human spine surgery, surgeon exposure is normally within recommended levels (33) but this could be influenced by the technique of use, the position of the source of radiation and the time taken to introduce each implant. In the present study we obeyed local radiation safety rules, including the use of personal lead aprons, thyroid protectors, and personal dosimeters (both attached to clothing and on finger rings) and there were no concerns on scheduled dose assessments after the study was completed. We kept exposure times as short as possible, although significantly more time is required to drill the holes in comparison to the FH technique. Exposure could be limited with rotation of the C-arm such that the primary beam is directed from dorsoventral oblique. Further study is required to assess radiation exposure for patient and surgeon with the EOFG technique presented.

There are several potential limitations to the study. Firstly, the small sample size and *ex-vivo* study design might not accurately reflect the accuracy and safety of the techniques in a clinical setting. We used drill-holes rather than inserting metallic implants, which might have had a tendency to under-estimate impingement of the vertebral canal and lateral cortex, given metallic implants might have been wider, which is particularly the case for 2.5 mm holes as screws with a larger outer diameter would most likely be employed on such cases. In addition, we only assessed the technique in the hands of experienced spinal surgeons who have already performed FH techniques on multiple occasions and felt comfortable with translating some skills into the EOFG technique. It might have been interesting to assess less experienced surgeons assuming the learning curve of the technique to be quite steep. Experience of viewing fluoroscopic images at an oblique angle may also take time to develop. Operating theaters are complex working environments with a draped patient, a range of surgical and anesthetic equipment and additional personnel which may influence the ability of the C-arm to freely move, orientate around the surgical table and expose the area of interest without obstruction. There is a requirement for specialized surgical tables with radiolucent portions which may not be suitable for patients of all sizes. We also widely reflected the para-spinal soft tissues which may have reduced the degree of tissues superimposed on the fluoroscopy images relative to clinical cases where vertebral fixation is being performed over a narrower area. From a technical perspective, there can also be challenges with re-aligning the C-arm with the partially inserted k-wire, if the C-arm has been “slid” away from the implant insertion point. This limitation could be overcome depending on the physical properties (shape and size) of the fluoroscopy detector plate, particularly if there was room to accommodate the detector and the drill. Lastly, it is not possible to “blind” surgeons to the technique they are performing, which might introduce bias in attempting a particular technique more accurately.

## 5 Conclusion

In conclusion, we developed the EOFG technique for safe drill hole placement in canine cadavers and found the technique to be significantly more accurate than traditional FH techniques. The EOFG technique shows promise for translation into a clinically setting, potentially improving surgeon confidence, implant purchase and construct strength, whilst reducing the likelihood of neurovascular injury and need for surgical revision.

## Data availability statement

The raw data supporting the conclusions of this article will be made available by the authors, without undue reservation.

## Ethics statement

Ethical approval for the study was obtained from the Primary Institutions Internal Review Committee (reference CVS-2024-004). Written informed consent was obtained from the individual(s) for thepublication of any identifiable images or data included in this article.

## Author contributions

CD: Conceptualization, Data curation, Formal analysis, Investigation, Methodology, Validation, Visualization, Writing – original draft, Writing – review & editing. VN: Conceptualization, Data curation, Formal analysis, Investigation, Methodology, Software, Validation, Visualization, Writing – review & editing. HT: Investigation, Methodology, Resources, Validation, Writing – review & editing. MN-C: Formal analysis, Investigation, Methodology, Writing – review & editing. JR: Conceptualization, Data curation, Formal analysis, Investigation, Methodology, Resources, Supervision, Validation, Writing – review & editing.

## References

[1] Selcer R Bubb W Walker T. Management of vertebral column fractures in dogs and cats: 211 cases (1977–1985). J Am Vet Med. (1991) 198:1965. 10.2460/javma.1991.198.11.1965 1874677

[2] Aikawa T Kanazono S Yoshigae Y Sharp NJH Munana KR. Vertebral stabilization using positively threaded profile pins and polymethylmethacrylate, with or without laminectomy, for spinal canal stenosis and vertebral instability caused by congenital thoracic vertebral anomalies. Vet Surg. (2007) 36:432–41. 10.1111/j.1532-950X.2007.00289.x 17614924

[3] Cabassu J Moissonnier P. Surgical treatment of a vertebral fracture associated with a haematogenous osteomyelitis in a dog. Vet Comp Orthop Traumatol. (2007) 20:227–30. 10.1160/VCOT-06-11-0089 17846691

[4] Dernell WS Van Vechten BJ Straw RC LaRue SM Powers E Withrow SJ. Outcome following treatment of vertebral tumors in 20 dogs (1986–1995). J Am Anim Hosp Assoc. (2000) 36:245–51. 10.5326/15473317-36-3-245 10825097

[5] Downes CJ Gemmill TJ Gibbons SE McKee WM. Hemilaminectomy and vertebral stabilisation for the treatment of thoracolumbar disc protrusion in 28 dogs. J Small Anim Pract. (2009) 50:525–35. 10.1111/j.1748-5827.2009.00808.x 19796311

[6] Hall DA Snelling SR Ackland DC Wu W Morton JM. Bending strength and stiffness of canine cadaver spines after fixation of a lumbar spinal fracture-luxation using a novel unilateral stabilization technique compared to traditional dorsal stabilization. Vet Surg. (2015) 44:94–102. 10.1111/j.1532-950X.2014.12268.x 25209367

[7] Garcia JNP MilthorpeI BK Russell D Johnson KA. Biomechanical study of canine spinal fracture fixation using pins or bone screws with polymethylmethacrylate. Vet Surg. (1994) 23:322–9. 10.1111/j.1532-950X.1994.tb00491.x 7839589

[8] Blass CE Seim Ill HB. Spinal fixation in dogs using Steinmann pins and methylmethacrylate. Vet Surg. (1984) 13:203–10. 10.1111/j.1532-950X.1984.tb00790.x

[9] McKee W Downes C. Vertebral stabilisation and selective decompression for the management of triple thoracolumbar disc protrusions. J Small Anim Pract. (2008) 49:536–9. 10.1111/j.1748-5827.2008.00582.x 18631222

[10] Zahn K Matis U. The clamp rod internal fixator-application and results in 120 small animal fracture patients. Vet Comp Orthop Traumatol. (2004) 17:110–20. 10.1055/s-0038-1632808

[11] Wheeler JL Cross AR Rapoff AJ. A comparison of the accuracy and safety of vertebral body pin placement using a fluoroscopically guided versus an open surgical approach: an *in vitro* study. Vet Surg. (2002) 31:468–74. 10.1053/jvet.2002.33616 12209418

[12] Driver CJ Lopez V Walton B Jones D Fentem R Tomlinson A. Instrumented cervical fusion using patient specific end-plate conforming interbody devices with a micro-porous structure in nine dogs with disk-associated cervical spondylomyelopathy. Front Vet Sci. (2023) 10:1208593. 10.3389/fvets.2023.1208593 37434865 PMC10331472

[13] Watine S Cabassu J Catheland S Brochier L Ivanoff S. Computed tomography study of implantation corridors in canine vertebrae. J Small Anim Pract. (2006) 47:651–7. 10.1111/j.1748-5827.2006.00070.x 17076788

[14] Gougeon E Meheust P. Pedicle screws implantation in polymethylmethacrylate construct to stabilise sixth lumbar vertebral body fracture in dogs: 5 cases (2015–2018). J Small Anim Pract. (2021) 62:1007–15. 10.1111/jsap.13400 34314046

[15] Jeffery ND. Vertebral fracture and luxation in small animals. Vet Clin North Am Small Anim Pract. (2010) 40:809–28. 10.1016/j.cvsm.2010.05.004 20732593

[16] Samer ES Forterre F Rathmann JMK Stein VM Precht CM Guevar J. Accuracy and safety of image-guided freehand pin placement in canine cadaveric vertebrae. Vet Comp Orthop Traumatol. (2021) 34:338–45. 10.1055/s-0041-1731808 34298579

[17] Hamilton-Bennett SE Oxley B Behr S. Accuracy of a patient-specific 3D printed drill guide for placement of cervical transpedicular screws. Vet Surg. (2018) 47:236–42. 10.1111/vsu.12734 29064584

[18] Guevar J Bleedorn J Cullum T Hetzel S Zlotnick J Mariani CL. Accuracy and safety of three-dimensionally printed animal-specific drill guides for thoracolumbar vertebral column instrumentation in dogs: bilateral and unilateral designs. Vet Surg. (2021) 50:336–44. 10.1111/vsu.13558 33340136

[19] Guevar J Samer ES Precht C Rathmann JMK Forterre F. Accuracy and safety of neuronavigation for minimally invasive stabilization in the thoracolumbar spine using polyaxial screws-rod: a canine cadaveric proof of concept. Vet Comp Orthop Traumatol. (2022) 35:370–80. 10.1055/s-0042-1750056 35760365

[20] Wheeler JL Lewis DD Cross AR Sereda CW. Closed fluoroscopic- assisted spinal arch external skeletal fixation for the stabilization of vertebral column injuries in five dogs. Vet Surg. (2007) 36:442–8. 10.1111/j.1532-950X.2007.00290.x 17614925

[21] Goffart LM Precht C Fosgate GT Maiolini A Hettlich BF. Accuracy of end-on fluoroscopy in predicting implant position in relation to the vertebral canal in dogs. Front Vet Sci. (2022) 9:982560. 10.3389/fvets.2022.982560 36337187 PMC9630941

[22] Xu R Ebraheim NA Ou Y Yeasting RA. Anatomic considerations of pedicle screw placement in the thoracic spine: Roy-Camille technique versus open-lamina technique. Spine. (1998) 23:1065–8. 10.1097/00007632-199805010-00021 9589548

[23] Belmont PJ Jr Klemme WR Dhawan A Polly DW Jr. *In vivo* accuracy of thoracic pedicle screws. Spine. (2001) 26:2340–6. 10.1097/00007632-200111010-00010 11679819

[24] Castro WH Halm H Jerosch J Malms J Steinbeck J Blasius S. Accuracy of pedicle screw placement in lumbar vertebrae. Spine. (1996) 21:1320–4. 10.1097/00007632-199606010-00008 8725923

[25] Guevara F Foss KD Harper TAM Moran CA Hague DW Hamel PES. *Ex vivo* comparison of pin placement with patient-specific drill guides or freehand technique in canine cadaveric spines. Vet Surg. (2024) 53:254–63. 10.1111/vsu.14042 37822110

[26] Mullins RA Espinel Ruperéz J Bleedorn J Hoey S Hetzel S Ortega C. Accuracy of pin placement in the canine thoracolumbar spine using a free-hand probing technique versus 3D-printed patient-specific drill guides: an *ex-vivo* study. Vet Surg. (2023) 52:648–60. 10.1111/vsu.13958 37071824

[27] Vaccaro AR Rizzolo SJ Allardyce TJ Ramsey M Salvo J Balderston RA. Placement of pedicle screws in the thoracic spine. Part I: morphometric analysis of the thoracic vertebrae. J Bone Joint Surg Am. (1995) 77:1193–9. 10.2106/00004623-199508000-00008 7642664

[28] Cinotti G Gumina S Ripani M Postacchini F. Pedicle instrumentation in the thoracic spine. Spine. (1999) 24:114–9. 10.1097/00007632-199901150-00003 9926379

[29] Elford JH Oxley B Behr S. Accuracy of placement of pedicle screws in the thoracolumbar spine of dogs with spinal deformities with three-dimensionally printed patient-specific drill guides. Vet Surg. (2020) 49:347–53. 10.1111/vsu.13333 31617955

[30] Mariani CL Zlotnick JA Harrysson O Marcellin-Little DJ Malinak K Gavitt A. Accuracy of three-dimensionally printed animal-specific drill guides for implant placement in canine thoracic vertebrae: a cadaveric study. Vet Surg. (2021) 50:294–302. 10.1111/vsu.13557 33373470

[31] Oxley B Behr S. Stabilisation of a cranial cervical vertebral fracture using a 3D-printed patient-specific drill guide. J Small Anim Pract. (2016) 57:277. 10.1111/jsap.12469 27004483

[32] Beer P Park BH Steffen F Smolders DLA Pozzi A Knell SC. Influence of a customized three-dimensionally printed drill guide on the accuracy of pedicle screw placement in lumbosacral vertebrae: an *ex vivo* study. Vet Surg. (2020) 49:977–88. 10.1111/vsu.13417 32255212

[33] Jones DP Robertson PA Lunt B Jackson SA. Radiation exposure during fluoroscopically assisted pedicle screw insertion in the lumbar spine. Spine. (2000) 25:1538–41. 10.1097/00007632-200006150-00013 10851103

